# An undifferentiated carcinoma at Klatskin-position with long-term complete remission after chemotherapy

**DOI:** 10.18632/oncotarget.25125

**Published:** 2018-04-24

**Authors:** Bruno Christian Köhler, Benjamin Goeppert, Nina Waldburger, Kai Schlamp, Peter Sauer, Dirk Jäger, Karl Heinz Weiss, Stephan Macher-Göppinger, Henning Schulze-Bergkamen, Peter Schirmacher, Christoph Springfeld

**Affiliations:** ^1^ Department of Medical Oncology, National Center for Tumor Diseases, University Hospital Heidelberg, Heidelberg, Germany; ^2^ Liver Cancer Center Heidelberg, University Hospital Heidelberg, Heidelberg, Germany; ^3^ Department of Pathology, University of Heidelberg, Heidelberg, Germany; ^4^ Department of Neuroradiology, University Hospital Heidelberg, Heidelberg, Germany; ^5^ Department of Gastroenterology, University Hospital Heidelberg, Heidelberg, Germany; ^6^ Institute of Pathology, University Medical Center Mainz, Mainz, Germany; ^7^ Department of Internal Medicine II, Marien-Hospital, Wesel, Germany

**Keywords:** cholangiocarcinoma, Klatskin tumor, undifferentiated carcinoma, chemotherapy, endoscopic retrograde cholangiography

## Abstract

**Background:**

Neoplasms anatomically adjacent to the bile duct usually derive from malignantly transformed cholangiocytes forming cholangiocarcinoma (CCA). CCAs are divided in extrahepatic (eCCA) and intrahepatic (iCCA) tumors. Patients with irresectable CCAs are treated with systemic chemotherapy and have an unfavorable prognosis with a median survival of about one year. Here, we report a case of an undifferentiated carcinoma in Klatskin-position with long-term remission after systemic chemotherapy.

**Case Presentation:**

A 65-year-old Caucasian male presented with painless jaundice caused by an undifferentiated carcinoma in Klatskin-position (Type IIIb). Alpha fetoprotein (AFP; 3675 IU/mL) and carbohydrate antigen 19-9 (CA 19-9; 183 U/ml) were elevated. An exploratory laparotomy was carried out, but the patient was found to be irresectable due to severe fibrosis caused by biliary obstruction. Histology showed an undifferentiated carcinoma with high proliferation rate, and the patient was therefore subjected to poly-chemotherapy treatment according to the FOLFOX6-protocol. During therapy, AFP decreased to normal. Subsequent CT scans and ERC revealed a complete remission. Four years past initial diagnosis, a new suspicious lesion in the liver is visible on MRT; however, AFP and CA 19-9 are still in the normal range.

**Conclusions:**

Our case demonstrates that histopathological defined diagnosis may significantly inform therapeutic decision-making in irresectable cholangiocarcinoma even in regard to conventional systemic therapy. In case of an undifferentiated carcinoma poly-chemotherapy may provide significant success.

## INTRODUCTION

Cholangiocarcinomas (CCA) are divided in three clinical phenotypes: intrahepatic CCA (iCCA) and extrahepatic CCA (eCCA) that can arise in perihilar (pCCA) or distal (dCCA) location. Especially relevant for a surgery-based treatment approach, pCCAs are further divided according to the Bismuth-Corlette classification in type I, II, III and IV. Type I and II are limited to the common hepatic duct. In type III tumors, the right (IIIa) or left (IIIb) hepatic ducts are involved [1, 2]. The tumor involves both hepatic ducts in type IV tumors. Even if recent molecular insights have paved the way for future tailored therapies in cholangiocarcinoma, surgery remains the only curative treatment option [3–5]. Due to relevant bile duct stenosis, cholestasis and subsequent painless jaundice may be the first clinical presentations [2, 6]. Achieving sufficient biliary drainage remains a major challenge in many patients. Patients with unresectable or metastasized CCAs are subjected to chemotherapy yielding overall survival of less than one year [7].

Even if the vast majority of malignant tumors arising in perihilar location are conventional adenocarcinomas of the pancreato-biliary type, other subtypes and patterns exist, which may influence therapeutic decision making. Here, we describe an interdisciplinary approach in a complex clinical case with an unusal histology demonstrating the importance of a resilient (histo-)pathological diagnosis to develop the best possible treatment, which in this case has led to an unexpected long-term remission after chemotherapy.

## CASE PRESENTATION

A 65-year-old male Caucasian patient was referred to our hospital with painless jaundice. The patient had a history of colon cancer (American Joint Committee on Cancer stage II, pT4, pN0, G2, M0, R0) 8 years ago treated with left hemicolectomy followed by adjuvant therapy with capecitabine. Thereafter, regular follow-up examinations did not show any tumor recurrence. Four years ago, a work-up due to elevated transaminases lead to the diagnosis of non-alcoholic steatohepatitis. No other chronic liver diseases were found.

**Figure 1:**
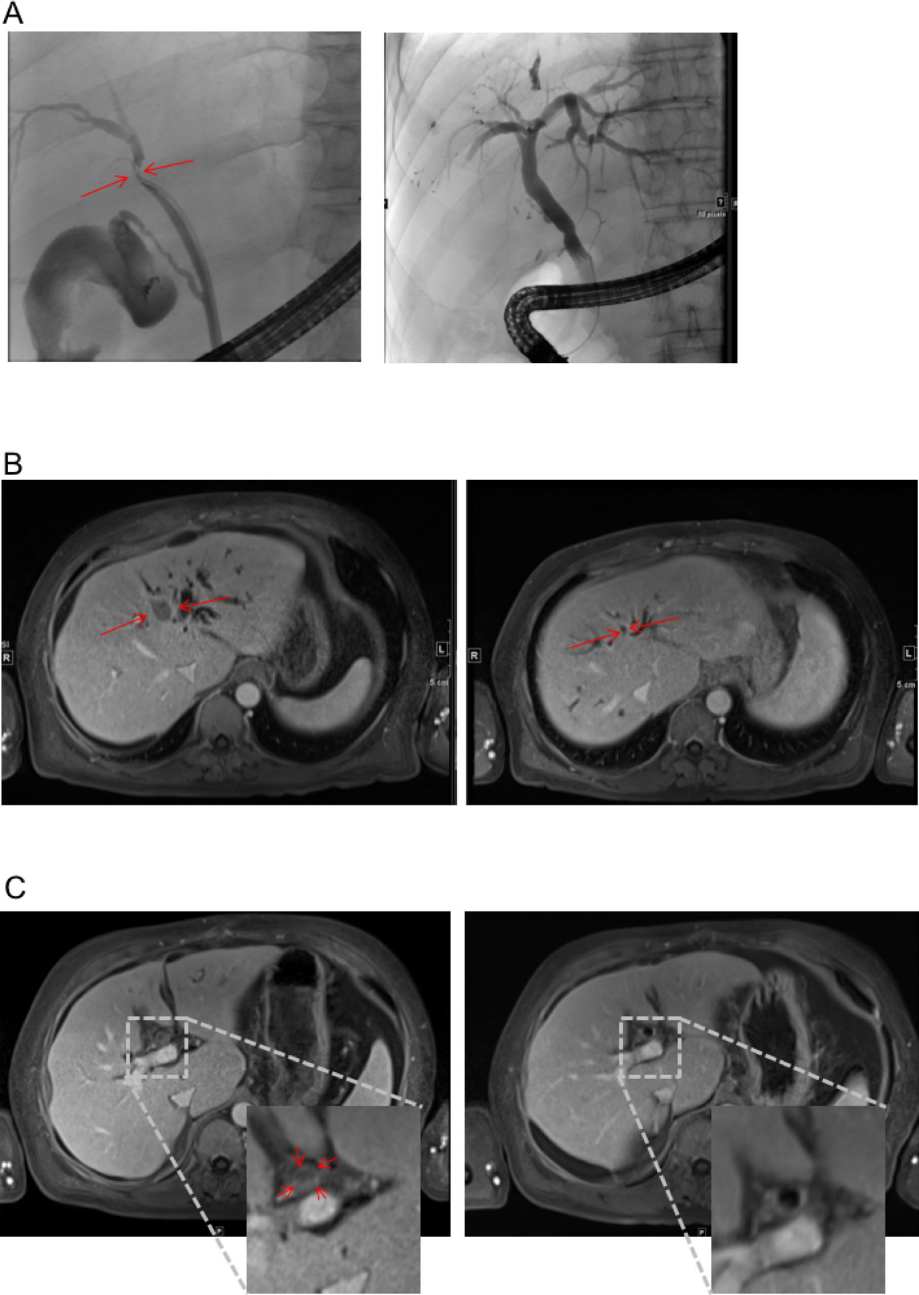
Response to therapy in MRI and ERC (**A**) ERC before placement of endoprothesis (left) and after successful therapy (right). Red Arrow heads indicate index lesion and relevant stenosis respectively. (**B** and **C**) MRI scan before (left) and after (right) 6 cycles of treatment according to FOLFOX6 regime. T1-weighted, Enlarged inset shows intraluminal tumor in the bile duct (C left).

**Figure 2:**
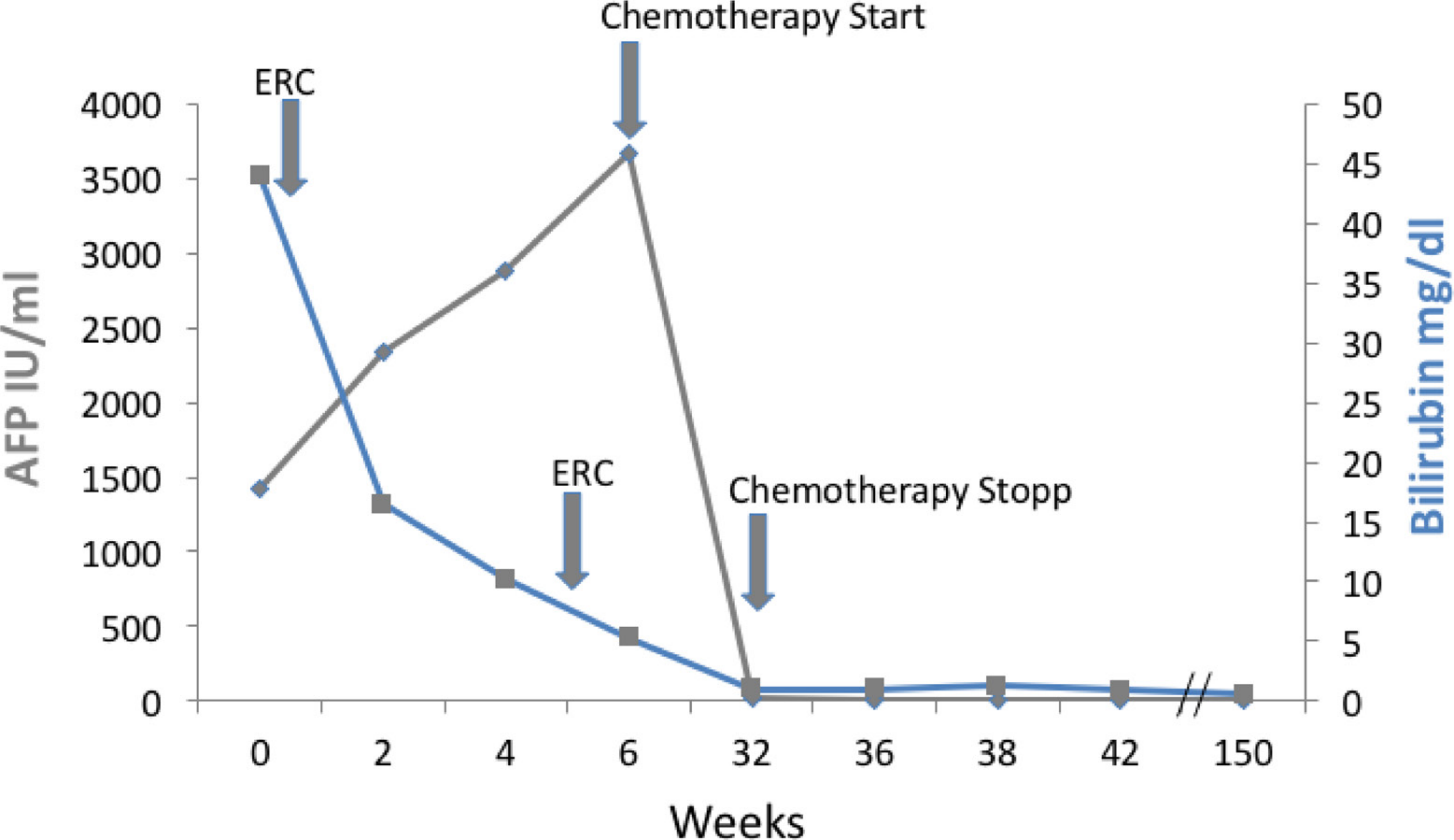
Course of bilirubin and AFP during treatment Shown are bilirubin and alpha fetoprotein (AFP) course from first referral. Bilirubin declined after ERC therapy, AFP declined to normal during therapy indicating a complete and sustained remission.

MRI-scan showed a malignant tumor mass in Klatskin-position, type IIIb according to Bismuth-Corlette classification (Figure 1) [8]. In addition, primarily suspicious lymph nodes were found at the hepatic hilum. No distant organ manifestations of the tumor were visible in MRI and CT-scans. Gastroscopy and colonoscopy were without pathological findings. At diagnosis, carcinoembryonic antigen (CEA) was in normal range with <0,2 µg/l, carbohydrate antigen (CA) 19-9 was elevated to 183 U/ml and alpha fetoprotein (AFP) was elevated to 3675 IU/ml (Figure 2).

Bilirubin was elevated to 38 mg/dl (Figure 2). Endoscopic retrograde cholangiography (ERC) showed an unaffected major duodenal papilla. Further examination of the biliary tree revealed a stenosis of the common hepatic duct and left hepatic duct with possible affection of the right hepatic duct, consistent with Bismuth-Corlette type IIIb/IV Klatskin-tumor (Figure 1A). Two plastic stents were implanted in the right and left hepatic duct. Subsequently, bilirubin dropped to 11.4 mg/dl (Figure 2).

An exploratory laparotomy aiming at a left hemihepatectomy in curative intention was then performed. An intraoperative liver biopsy showed severe chronic cholestatic and biliary fibrosis with septa formation compatible with chronic biliary obstruction. Two suspicious lymph nodes taken from the liver hilum showed reactive changes but no metastases. Due to the intraoperatively confirmed severe septal fibrosis, tumor resection was not conducted because of the risk of hepatic failure.

A second ERC after surgery showed persistent dilatation of the prestenotic bile ducts. When a balloon retrieval catheter was used in an attempt to clean the bile ducts, coagulated blood and tissue was retrieved and sent to pathology. New plastic stents were inserted. Afterwards, bilirubin further dropped to 1.8 mg/dl (Figure 2). Stents were regularly exchanged every 3 months in follow up ERCs during the further course of treatment.

**Figure 3:**
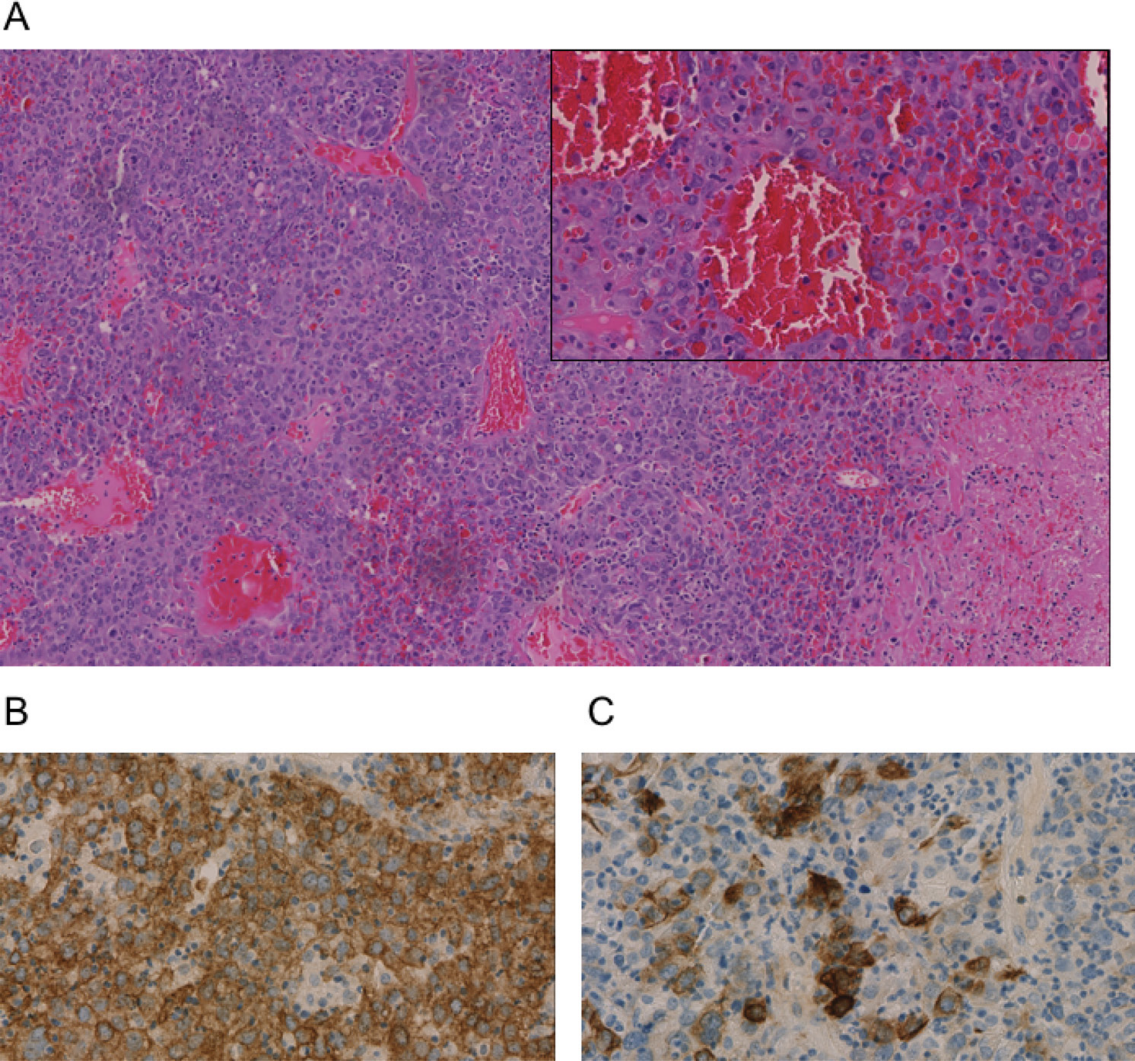
Immunohistochemistry of an undifferentiated carcinoma in Klatskin-position All stainings were performed on a biopsy taken during ERC after paraffin embedding. (**A**) Hematoxylin and Eosin (H&E), (**B**) pan-cytokeratin marker AE1/3 (**C**) CK20 staining. Representative images are shown.

The tissue obtained from the bile duct during ERC showed formations of a partially necrotic solid and invasively growing malignant tumor with strong tumor-associated inflammation composed of a quite homogenous large-sized tumor cells. Immunohistochemistry showed positivity of the tumor cells for broad spectrum Cytokeratin (AE1/3) as well as partially for Cytokeratins (CK) 18, 19 (∼60% of tumor cells), and 20 (Figure 3). All other markers aiming at a further lineage differentiation were negative (Synaptophysin, Vimentin, Desmin, ASMA, S100, CD34, OCH1E5, Arginase, CK7, TTF1, Napsin, CDX2, CK5/6, CA19-9, AFP, p63, Uroplakin 3, BerEp 4, PLAP, CD45, and CD30) (Figure 3). Therefore, an undifferentiated liver carcinoma was diagnosed. Tumor proliferation, measured by Ki-67 immunohistology, was high (40–70%, integral 60%).

Due to its inoperability, undifferentiated histology, and high proliferation rate, it was decided by an interdisciplinary tumor board to treat the patient with chemotherapy. Since bilirubin was still elevated, the FOLFOX6 protocol (fluorouracil 400 mg/m^2^ IV bolus, followed by 46-hour 5-FU infusion [2400 mg/m^2^], leucovorin [400 mg/m^2^], and oxaliplatin [100 mg/m^2^]) that can also be administered in patients with hyperbilirubinemia was chosen [9]. The initial dose was reduced (50%), and therapy was well tolerated by the patient. The patient’s bilirubin dropped into normal range, and a second cycle was given at full intended dose. Therapy according to the FOLFOX6 protocol was administered for 3 months (Figure 2). During therapy, AFP concentration dropped to 12.8 IU/ml and CA 19-9 level to 7.4 U/ml within 24 weeks. MRI-scan showed a marked shrinkage of the intrahepatic tumor mass, dilatation of prestenotic bile ducts was relieved (Figure 1B and 1C). Lymph nodes at the portal vein, the liver hilus and the mesentery remained enlarged, and there was some ascites. 16 months after start of chemotherapy, the biliary plastic stents were removed since the biliary stenoses had disappeared.

During the further course, AFP and CA19-9 stayed in the normal range. 48 months after diagnosis and 42 months after termination of chemotherapy, the patient is in excellent condition (ECOG 0, Karnofsky index 100%). Bilirubin levels stayed in the upper normal range around 1 mg/dl (Figure 2). Finally, in the MRI 48 months after diagnosis, a suspicious lesion in the liver was observed, but AFP was still in the normal range. Further follow-up by MRI is planned.

## DISCUSSION

Among epithelial liver tumors, undifferentiated tumors are rare, accounting for less than 2% of neoplasms [10, 11]. Etiology, driving mechanisms and cell of origin are largely unknown, and are likely to vary among cases. Anatomically, most reported cases of hepato-biliary tumors show an intrahepatic tumor localization. Here, we report the first case in Klatskin-position [12, 13]. The vast majority of Klatskin tumors represent adenocarcinomas of the conventional pancreato-biliary type. Other, rare histologies include squamous cell carcinoma, adenosquamous carcinomas and

neuroendocrine tumors [10, 14–16]. An undifferentiated carcinoma may arise from hepatocytes, biliary epithelial cells or potential precursor cells. In our case, growth and position argue for an origin from the biliary epithelium, but this could not be proven in the tumor biopsy and origin from hepatocytes with secondary infiltration of the bile duct cannot be completely excluded. Immunohistology gave some indication of adeno-differentiation (CK18 in the absence of CK5/6) but signs of biliary (only partial CK19 positivity) and hepatocellular differentiation (only serum AFP elevation, no demonstrable hepatocyte marker or AFP in the biopsy) were weak and inconclusive. Therefore, the case has to be classified as an undifferentiated primary liver carcinoma.

Literature regarding undifferentiated carcinomas is scarce, therapeutic guidelines or recommendations are not available [17–19]. Undifferentiated carcinomas have been described in a variety of anatomical localizations including stomach, small intestine and pancreas. In the available case reports, therapeutic approaches vary, but adhere in most cases to standard treatment for a typically differentiated carcinoma in the corresponding anatomical localization. For instance, S1 (Tegafur^®^) and Cisplatin have been used as treatment for undifferentiated gastric cancer in a Japanese case series. This treatment approach seems to be ineffective, since all patients had no disease control [17]. In contrast, undifferentiated carcinomas of the pancreas have been successfully treated with Gemcitabine leading to a long-term remission [18]. Those few reports with contrary outcome emphasize the complexity and singularity of undifferentiated carcinomas probably depending on the affected organ system, proliferation and cell of origin.

Due to the rarity of undifferentiated liver carcinomas, no therapeutic recommendations are established. We have previously shown the efficacy of 5-fluoropyrimidine based chemotherapy for highly proliferative liver tumors with stem/progenitor features [20]. Even if the best therapy regime remains elusive and larger prospective trials for rare liver tumors are urgently needed, 5-FU appears to be an effective backbone for chemotherapy. Although the current case lacked markers of stem/progenitor cells it shared AFP expression, undifferentiated histology, and high proliferation rate (>50%) with our previous case series. Based on this rationale, the patient was treated with poly-chemotherapy. Intriguingly, only 6 six courses of chemotherapy according to the FOFLFOX6-protocol resulted in long-term remission for about 4 years. Even if the now suspected relapse can be confirmed, the patients survived much longer than patients with cholangiocarcinomas treated with the current standard therapy with cisplatin and gemcitabine. Interestingly, the pretreatment tumor biopsy showed strong tumor associated inflammation. Thus, it may be speculated in analogy to observations in metastatic colorectal cancer, that intense tumor-associated inflammatory cell infiltration may support tumor elimination by chemotherapy and may be an additional indicator of favourable response to chemotherapy [21, 22].

## CONCLUSIONS

To our knowledge, the presented case represents the first reported case of an undifferentiated carcinoma in Klatskin-position. Our multidisciplinary approach guided by the peculiar histopathological phenotype has opened a window for a successful treatment rationale: i) A biopsy may provide therapeutically relevant information even in an unresectable conventionally appearing Klatskin tumor ii) undifferentiated liver carcinomas may be exceptionally sensitive towards (poly)-chemotherapy, which may argue in favor of chemotherapy as the first and most effective treatment modality in these cases, and iii) tumor-associated inflammation should be explored as a predictor of good chemotherapy response in primary liver cancer.
